# Biphasic *in vitro* maturation with C-type natriuretic peptide enhances the developmental competence of juvenile-goat oocytes

**DOI:** 10.1371/journal.pone.0221663

**Published:** 2019-08-23

**Authors:** Sandra Soto-Heras, Irene Menéndez-Blanco, Maria-Gracia Catalá, Dolors Izquierdo, Jeremy G. Thompson, Maria-Teresa Paramio

**Affiliations:** 1 Departament de Ciència Animal i dels Aliments, Facultat de Veterinària, Universitat Autònoma de Barcelona, Bellaterra, Barcelona, Spain; 2 Robinson Research Institute, School of Paedriatics and Reproductive Health, The University of Adelaide, Adelaide, South Australia, Australia; 3 Adelaide Medical School, The University of Adelaide, Adelaide, South Australia, Australia; 4 Australian Research Council Centre of Excellence for Nanoscale BioPhotonics and Institute for Photonics and Advanced Sensing, Davies Research Centre, The University of Adelaide, Adelaide, South Australia, Australia; China Agricultural University, CHINA

## Abstract

In vitro embryo production success in juvenile animals is compromised due to their intrinsic lower oocyte quality. Conventional in vitro maturation (IVM) impairs oocyte competence by inducing spontaneous meiotic resumption. A series of experiments were performed to determine if maintaining meiotic arrest during a pre-maturation culture phase (pre-IVM) prior to conventional IVM improves oocyte competence of juvenile-goat (2 months old) cumulus-oocyte complexes (COCs). In experiment 1, COCs were cultured with C-type natriuretic peptide (CNP; 0, 50, 100, 200 nM) for 6 and 8 h. Nuclear stage was assessed, revealing no differences in the incidence of germinal vesicle (GV) breakdown. In experiment 2, the same CNP concentrations were assessed plus 10 nM estradiol, the known upstream agonist activating expression of NPR2, the exclusive receptor of CNP. CNP (200 nM) plus estradiol increased the rate of oocytes at GV stage at 6 h compared to control group (74.7% vs 28.3%; P<0.05) with predominantly condensed chromatin configuration. In experiment 3, relative mRNA quantification revealed *NPR2* expression was down-regulated after pre-IVM (6 h). In experiment 4, analysis of transzonal projections indicated that pre-IVM maintained cumulus-oocyte communication after oocyte recovery. For experiments 5 and 6, biphasic IVM (6 h pre-IVM with CNP and estradiol, plus 24 h IVM) and control IVM (24 h) were compared. Biphasic IVM increased intra-oocyte glutathione and decreased ROS, up-regulated DNA-methyltransferase 1 and pentraxin 3 expression and led to an increase in rate of blastocyst development compared to control group (30.2% vs 17.2%; P<0.05). In conclusion, a biphasic IVM, including a pre-IVM with CNP, maintains oocyte meiotic arrest for 6 h and enhances the embryo developmental competence of oocytes from juvenile goats.

## Introduction

Juvenile in vitro embryo transfer (JIVET) has great potential for improving breeding programs as it can increase the rate of genetic gain by reducing the generation interval [1], especially as ovaries from juvenile females provide higher numbers of oocytes than adults [2]. However, oocytes retrieved from small follicles (< 3 mm) developed to the blastocyst stage at a lower rate than oocytes from adult females (reviewed by Paramio & Izquierdo [3]).

Oocyte in vitro maturation (IVM) is a limiting step for in vitro embryo production (IVEP). Conventional IVM impairs oocyte competence (the ability to sustain embryo development and lead a pregnancy to term [4]). Competence acquisition depends on changes at nuclear and cytoplasmic levels that occur during folliculogenesis prior to final oocyte maturation (reviewed by Gilchrist & Thompson [5]). But oocytes spontaneously resume meiosis in vitro following retrieval from antral follicles [6], which prevents the oocyte to fulfill this process.

High intra-oocyte cyclic AMP levels sustain meiotic arrest [7] by preventing the activation of the maturation-promoting factor [8]. The follicle maintains high cAMP via C-type natriuretic peptide (CNP) and its receptor (NPR2). CNP increases cGMP levels in cumulus cells (CC) and oocytes [9,10] which inhibits phosphodiesterase 3A (PDE3A), the main cAMP hydrolyzing enzyme [11]. After COC are isolated from the follicle, PDE3A is released from cGMP inhibition, which causes a rapid cAMP decrease and further meiosis resumption [12].

Novel cAMP-mediated IVM systems can improve oocyte maturation and competence (reviewed by Gilchrist et al. [8]). One such system is a biphasic IVM that consists of a pre-maturation (pre-IVM) phase using a PDE inhibitor, which maintains meiotic arrest by preventing cAMP degradation, followed by a conventional IVM period. The physiological cAMP modulator in the ovary, CNP, can be used as a PDE inhibitor for biphasic IVM, instead of other synthetic molecules such us 3-Isobutyl-1-methylxanthine (IBMX; a non-specific PDE inhibitor). The pre-IVM culture with CNP can improve blastocyst development in different animal species [10,13–16], including adult goat after parthenogenetic activation [17]. Some reports indicate that pre-IVM with CNP could even overcome the limitations of IVEP with low-competence oocytes, such as those from juvenile animals. In juvenile mice, oocytes cultured in a biphasic IVM system with CNP developed to the blastocyst stage at comparable rate to in vitro fertilization (IVF) of ovulated oocytes [18]. In women, biphasic IVM increased the rate of oocytes from small antral follicles that develop to good-quality blastocysts, compared to conventional IVM [19].

The success of cAMP-modulated IVM has been related to the maintenance of gap junction communication (GJC) between CC and oocytes [20,21]. Maintaining high cAMP increases intra-oocyte glutathione (GSH) levels [21,22], a well-known parameter associated with oocyte competence [23,24]. This likely benefits juvenile oocytes, which are more sensitive to reactive oxygen species (ROS) due to lower GSH synthesis [25]. Mitochondrial number and activity are also enhanced [10,22], which are additionally related to oocyte competence [26,27]. Lastly, cAMP-modulated IVM up-regulates genes involved in cell communication and metabolism, steroidogenesis and formation of extracellular matrix in cumulus cells [28], all of which are likely to reflect improved oocyte competence.

We hypothesized that biphasic IVM could improve the in vitro production of transferable embryos in juvenile goats. The aim of this study was to design a pre-IVM using CNP that sustains meiotic arrest and CC-oocyte communication, providing the oocyte with additional embryo developmental competence.

## Materials and methods

Unless indicated all chemicals were purchased from Sigma-Aldrich Chemical Co (St. Louis, USA).

### Animal ethics

The approval by the Ethics Committee on Animal and Human Experimentation (CEEAH) of the Autonomous University of Barcelona was not required as the experiments were performed under in vitro conditions. Ovaries were collected from goats of murciano-granadina breed at *Gremial de Catalunya SA* slaughterhouse in Castellbisbal, Barcelona, Spain.

### Oocyte recovery

Ovaries from juvenile goats (1 to 2 months old) were obtained at a local slaughterhouse and maintained at 35–37°C in phosphate buffered saline (PBS). COCs were recovered by slicing of the ovary in HEPES-buffered (25 mM) TCM-199 supplemented with 2.2 mg/mL NaHCO_3_, 50 mg/mL gentamycin and 11.1 mg/mL heparin. In pre-IVM experimental groups the slicing medium was further supplemented with 500 μM IBMX to avoid meiotic resumption during oocyte recovery. Oocytes with at least two layers of compact CC and homogeneous cytoplasm were selected.

### Oocyte in vitro maturation

Oocytes were cultured in pre-IVM, pre-IVM plus IVM (biphasic IVM) and IVM depending on the experiment.

#### Pre-IVM

COCs were cultured in 100-μL drops of pre-IVM medium covered with mineral oil (25–30 COCs / 100 μL) for 6 or 8 h at 38.5°C in humidified air with 5% CO_2_. Basic pre-IVM medium was TCM-199 with 4 mg/mL bovine serum albumin (BSA), 0.2 mM sodium pyruvate, 1 mM glutamine, 100 μM cysteamine and 5 μg/mL gentamycin. The basic medium was supplemented with CNP (50, 100, 200 nM) and 10 nM 17β-estradiol (E2), depending on the experimental group.

#### IVM

COCs were cultured in drops of IVM medium covered with mineral oil (25–30 COCs / 100 μL) for 24 h at 38.5°C in humidified air with 5% CO_2_. IVM medium was adapted from Catalá et al. [29]: TCM-199 supplemented with 5 μg/mL LH, 5 μg/mL FSH, 3.7 μM E2, 10 ng/mL epidermal growth factor (EGF), 0.2 mM sodium pyruvate, 1 mM glutamine, 10% (v/v) fetal bovine serum (FBS) and 5 μg/mL gentamycin.

### Assessment of oocyte nuclear stage

Oocytes were stained with orcein, adopted from Prentice-Biensch et al. [30]. Briefly, oocytes were fixed in ethanol:acetic (3:1) overnight at 4°C, mounted in a slide, covered with a wax supported coverslip and stained with 1% orcein (w/v) in 45% acetic acid solution (v/v). Nuclear stage was assessed with a phase-contrast microscope (Olympus BX50) and classified as: germinal vesicle (GV), germinal vesicle breakdown (GVBD), metaphase I (MI) and metaphase II (MII). GVs were classified according to chromatin configuration and nucleolus size as described by Sui et al. [31]: GV1 (large nucleolus and diffuse filamentous chromatin), GV net-like (condense net-like chromatin), GV clumped (condense clumped chromatin).

### Assessment of transzonal projections

Transzonal projections (TZPs) were evaluated as described by Soto-Heras et al. [32]. COCs were partially denuded and fixed in 4% paraformaldehyde (PF; w/v) for 20 min at 38°C. COCs were permeabilized with 0.25% Triton X-100 in PBS (v/v) for 30 min and incubated with 5 μg/mL phalloidin-FITC in 0.4% BSA-PBS (w/v) for 60 min at room temperature (RT). COCs were counterstained with 1 μg/mL Hoechst 33258 (Invitrogen, Eugene, OR, USA) for 10 min. COCs were mounted in a poly-L-lysine-treated coverslip with a drop of Vectashield mounting medium (Vector Laboratories, Burlingame, CA, USA) within a reinforcement ring, and kept at -20°C until analysis with confocal laser microscopy (Spectral Leica TCSSP5, Mannheim, Germany). Images were taken with 63X magnification under mineral oil (phalloidin-FITC: λ_ex_ 488 nm, λ_em_ 520 nm; Hoechst: λ_ex_ 405 nm, λ_em_ 465 nm). TZPs were observed as continuous filaments going from CC to the oocyte. TZP density was quantified with ImageJ software (Version 1.51h; National Institute of Health, Bethesda MD, USA) by measuring average pixel intensity in the zona pellucida area delimited by polygon selection tool.

### Assessment of reactive oxygen species and glutathione levels

Intra-oocyte ROS level was measured by staining with 2’,7’-dichlorodihydrofluorescein diacetate (H_2_DCF-DA; Molecular Probes Inc., Eugene, OR, USA), adopted from Park et al. [33]. Intra-oocyte GSH content was measured with monochlorobimane (MCB), adopted from Keelan et al. [34]. Briefly, oocytes were denuded and incubated for 15 min either with 10 μM H_2_DCF-DA or 12.5 μM MCB in 0.4% BSA-PBS at 38.5°C. Oocytes were washed in 0.1% BSA-PBS, immediately transferred with a 10-μL drop to a slide and observed under Olympus BX50 epi-fluorescent microscope with 10X magnification (H_2_DCF-DA: λ_ex_ 460 nm, λ_em_ 525 nm; MCB: λ_ex_ 370 nm, λ_em_ 461 nm). Average fluorescence intensity per oocyte was measured with Image J software and normalized to the background average intensity.

### RNA relative quantification

RNA relative quantification was performed for genes listed in Table 1. Groups of 10 COCs were washed in PBS with 0.3% polyvinylpyrrolidone (PVP, w/v), snap-frozen in buffer RLT (Qiagen RNeasy mini kit; Qiagen, Ambion Inc., Austin TX, USA) and kept at -80°C until analysis. RNA was extracted with Qiagen RNeasy mini kit and eluded in 30 μL RNase-free water. RNA concentration was assessed with Qubit^™^ RNA HS assay kit (Thermo Fisher Scientific, Waltham MA, USA) and RNA integrity (RIN) with Agilent RNA 6000 pico chip on Agilent 2100 bioanalyzer (Agilent technologies, Waldbronn, Germany). DNase treatment was performed with Turbo DNA-free kit (Applied biosystems, Foster City CA, USA) prior to reverse transcription (RT). RT was performed with High-Capacity cDNA Reverse Transcription Kit (Applied biosystems) in 30 μL reaction volume, and a Bio rad T100 thermal cycler (Bio Rad Laboratories, Hercules CA, USA) set to 25°C 10 min, 37°C 120 min and 95°C 5 min. For real-time quantitative PCR (RT-qPCR), SYBR^®^ Select Master Mix (Applied Biosystems) was used with 15 μL reaction volume. RT-qPCR was performed in Quant Studio 12K Flex Real-rime PCR system (Applied biosystems). Thermo-cycling conditions consisted of an initial holding (50°C 2 min) then denaturation (95°C 10 min) steps, amplification stage (95°C 15 s and 60°C 1 min, repeated 40 cycles), and final melting curve (95°C 15 s, 60°C 1 min, 95°C 15 s). Three replicates for each sample and primer were performed. Prior to final analysis, a standard curve was done for each gene to determine the PCR efficiency (80–110%). RNA was quantified with the 2^-ΔΔCT^ method described by Livak & Schmittgen [35] in relation to two reference genes and the reference group. The RT app on Thermo Fisher cloud was used for this calculation.

**Table 1:** Primer detailed information for each gene analyzed.

**Gene**	**Sequence (5’-3’)**	**GenBank accession no.**	**Fragment size (bp)**
*RPL19*	Forward: AGATTGACCGCCACATGTATCAC	NC_030826.1	79
	Reverse: TCCATGAGAATCCGCTTGTTTT		
*RPS9*	Forward: ACAAACGTGAGGTCTGGAGGG	NC_030825.1	88
	Reverse: GGGTCTTTCTCATCCAGCGTC		
*DNMT1*	Forward: GGTGAAAAGGCTCTTCTTGGC	NC_022299.1	83
	Reverse: AATAGTGGTGCGTACTCTGGGC		
*GDF9*	Forward: TCTACAACACTGTTCGGCTCTTCA	NC_022299.1	122
	Reverse: CACAACAGTAACACGATCCAGGTT		
*BMP15*	Forward: TCGGGTACTATACTATGGTCTCAATTC	NW_017189516.1	141
	Reverse: GCCTCAATCAGAAGGATGCTAATGG		
*NPR2*	Forward: TCTGTACGCCGAAGTCCTGAA	NC_030815.1	87
	Reverse: CGTCCTTGCATCTTCTCGACA		
*PTX3*	Forward: TGGACAACGAAATAGACAATGGAC	NC_030808.1	76
	Reverse: TCGGAGTTCTCACGACTGCA		
*TNFAIP6*	Forward: GGAATCCGTCTCAATAGAAGTGAAA	NC_030809.1	81
	Reverse: TGTAAACACACCACCACACTCCTT		
*FSHR*	Forward: GTTTTGAAAGTATGATTGTATGGCTGAG	NC_030818.1	80
	Reverse: GAGTTGGGTTCCATTGAATGC		

Reference genes: *RPL19* (ribosomal protein L19), *RPS9* (ribosomal protein S9); Quantified genes: *DNMT1* (DNA methyltransferase 1), *GDF9* (growth-differentiation factor 9), *BMP15* (bone morphogenetic protein 15), *NPR2* (natriuretic peptide receptor 2), *PTX3* (pentraxin 3), *TNFAIP6* (TNF alpha induced protein 6), *FSHR* (follicle stimulating hormone receptor).

### Quantification of mtDNA copy number

Mitochondrial DNA was quantified with qPCR as described by Lamas-Toranzo et al. [26], using primers GTTAAACGGCCGCGGTATTC (forward) and TCACCCCAACCAAAACTGCT (reverse) that amplify a 262 bp specific product from goat mitochondrial DNA (GenBank accession no: NC_005044.2). Oocytes were completely denuded and the zona pellucida was removed with 0.5% protease from *Streptomyces griseus* in 0.3% PVP-PBS (w/v). Oocytes were individually placed in 0.2-ml tubes, snap frozen and stored at -80°C until analysis. Oocytes were digested with 8 μl PicoPure DNA extraction kit (Applied Biosystems) by incubating at 65°C for 1 h followed by inactivation at 95°C for 10 min. A standard curve was created by cloning the specific goat mitochondrial product into the vector pMD20 (Takara, Kusatsu, Japan). Quantitative PCR was performed with Gotaq qPCR Master Mix (Promega, Madison WI, USA) on a MIC quantitative thermo-cycler (Biomolecular Systems, Upper Coomera, Australia). Thermo-cycling conditions consisted in initial denaturation step (95°C 5 min), amplification step (94°C 15 s, 56°C 30 s, 72°C 20 s, repeated 40 cycles), and a final melting curve. DNA was quantified following the comparative quantification cycle method as described by Bermejo-Álvarez et al. [36]. Briefly, the Ct value was determined in the region of the amplification curve where increasing one cycle was equivalent to doubling the amplified PCR product. The ΔCt was normalized by subtracting from each Ct value the highest average Ct of the experimental groups, i.e. the average Ct of the group with the lowest mtDNA number. Fold changes were determined using the 2^-ΔΔCT^ formula.

### In vitro embryo production

After IVM, COCs were co-cultured with 4 x 10^6^ sperm/mL in 100-μL drops of BO-IVF medium (IVF Bioscience, Falmouth, United Kingdom) covered with mineral oil (15 COCs / 100 μL) at 38.5°C in humidified air with 5% CO_2_. Frozen sperm from two bucks was thawed at 36°C for 1 min and selected with BoviPure density gradient (Nidacon EVB S.L., Barcelona, Spain) by centrifuging for 20 min at 250 X g. After 20 h of IVF, presumptive zygotes were denuded and cultured in 10-μL drops of BO-IVC medium (IVF Bioscience) covered with Nidoil (Nidacon, Mölndal, Sweden) (8–10 zygotes / drop) at 38.5°C in humidified air with 5% CO_2_ and 5% O_2_. Cleavage was recorded at 48 h post-fertilization (hpf) and blastocyst rate at 8 days post-fertilization (dpf).

### Assessment of blastocyst quality

Blastocysts were stained as described by Thouas et al. [37]. Briefly, blastocysts were incubated in TCM-199 with 1% Triton X-100 (v/v) and 100 μg/mL propidium iodide (PI) for 25 s and transferred to pure ethanol with 25 μg/mL Hoechst 33258, where they were kept at 4°C overnight. Blastocysts were mounted with a drop of glycerol and observed under Olympus BX50 epifluorescence microscope (λ_ex_ 370 nm, λ_em_ 465 nm). Differential cell count was performed with Image J software. Cells protected from triton permeabilization and stained only with Hoechst (blue) were assumed to be inner cell mass (ICM); cells permeabilized with triton and stained with PI (red) were assumed to be trophectoderm (TE).

### Experimental design

#### Experiment 1

**Effect of pre-IVM with C-type natriuretic peptide on meiotic arrest.** COCs were cultured in pre-IVM medium supplemented with 0 (control), 50, 100 or 200 nM CNP for 6 or 8 h. At the end of each culture period, nuclear stage was evaluated. A total of 47–48 oocytes were assessed per treatment and time point (four replicates).

#### Experiment 2

**Effect of pre-IVM with C-type natriuretic peptide and estradiol on meiotic arrest.** COCs were cultured in pre-IVM medium supplemented with CNP and E2: 0 (control), E2 (10 nM), 50 nM CNP + E2, 100 nM CNP + E2, or 200 nM CNP + E2. At the end of the culture period (6 or 8 h), nuclear stage and GV chromatin configuration were evaluated. A total of 46–50 oocytes were assessed per treatment and time point (four replicates).

#### Experiment 3

**Effect of pre-IVM on the expression of natriuretic peptide receptor 2.** Relative expression of *NPR2* was analyzed in COCs after 6 h of pre-IVM with 200 nM CNP, or 200 nM CNP + 10 nM E2. An additional group was tested: uncultured COCs frozen after recovery from the ovary (control 0 h). A total of five samples (10 COCs / sample) were analyzed per group.

#### Experiment 4

**Effect of pre-IVM with C-type natriuretic peptide and estradiol on cumulus-oocyte communication.** A biphasic IVM system was compared to control IVM. For biphasic IVM, COCs were cultured for 6 h in pre-IVM medium with 200 nM CNP plus 10 nM E2, followed by 24 h IVM. For control IVM, oocytes were cultured for 24 h in IVM medium. COCs were fixed and stained with phalloidin-FITC at different time points: after oocyte recovery (Control 0 h), 6 h pre-IVM, 6 h IVM, 6 h pre-IVM + 24 h IVM, 24 h IVM. A total of 32–46 COCs were assessed per condition (five replicates).

#### Experiment 5

**Effect of biphasic IVM (6 h pre-IVM plus 24 h IVM) on oocyte quality.** Two experimental groups were tested: biphasic IVM (6 h pre-IVM with 200 nM CNP plus 10 nM E2, followed by 24 h IVM) and control IVM (24 h). Different parameters related to oocyte quality were assessed: nuclear stage (46–47 oocytes per group, four replicates), GSH levels (30 oocytes, three replicates), ROS levels (30 oocytes, three replicates), mtDNA copy number (30 oocyte, three replicates), and relative mRNA quantification of *DNMT1*, *GDF9*, *BMP15*, *PTX3*, *TNFAIP6* and *FSHR* (five samples, 10 COCs / sample). For mtDNA copy number and mRNA quantification COCs were also analyzed after follicle recovery (control 0 h).

The following RNAs were selected for quantification due to their relation to acquisition of oocyte competence during follicular development: DNMT1 (reviewed by Uysal & Ozturk [38]), GDF9 and BMP15 (reviewed by Gilchrist et al. 2008 [39]), and FSHR (reviewed by Ferreira et al. [40]). PTX3 and TNFAIP6 are extracellular matrix proteins promoted by EGF signaling during oocyte maturation (reviewed by Brown et al. [41]).

#### Experiment 6

**Effect of biphasic IVM (6 h pre-IVM plus 24 h IVM) on embryo development.** Two experimental groups were tested: biphasic IVM (6 h pre-IVM with 200 nM CNP plus 10 nM E2, followed by 24 h IVM) and control IVM (24 h). After each IVM system, COCs were fertilized and embryo cultured. A total of 193–196 oocytes were cultured per group (five replicates). Expanded and hatched blastocysts were stained (22–33 blastocysts per group, four replicates).

### Statistical analyses

Nuclear stage, embryo production, blastocyst cell number, ROS levels, GSH levels and TZPs density were analyzed with two-way ANOVA followed by Tukey’s multiple-comparison test. Treatment was set as the fixed factor and replicate as the random variable. Data from nuclear stage and embryo development did not present a normal distribution and were square root arcsine transformed prior to ANOVA. Normality and homogeneity of variance were reassessed and confirmed after transformation. SAS/STAT^®^ software version 9.4 (SAS institute Inc., Cary, NC, USA) was used for these analyses. The mtDNA copy number was analyzed by one-way ANOVA using the SigmaStat software (Jandel Scientific, San Rafael, CA). The relative mRNA quantification was analyzed with the RT app on Thermo Fisher cloud using a model of integrated correlation. Results were considered statistically significant when P<0.05.

## Results

## Pre-IVM with C-type natriuretic peptide maintains meiotic arrest in the presence of estradiol, with predominant condensed clumped chromatin configuration (experiments 1, 2 and 3)

**Fig 1:**
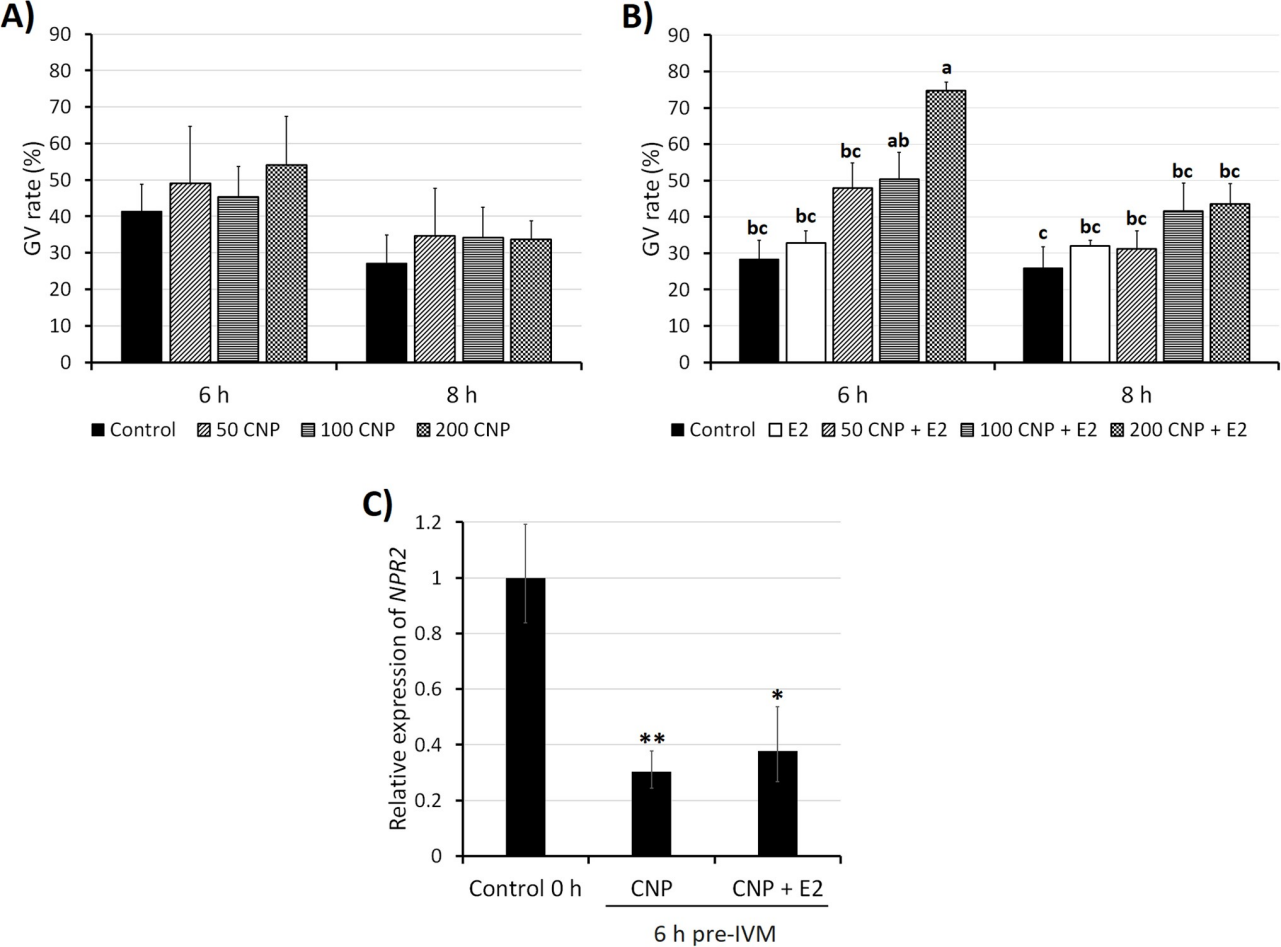
Effect of pre-IVM with CNP and estradiol on the maintenance of meiotic arrest in juvenile-goat oocytes. (A) Germinal vesicle (GV) rate of oocytes cultured for 6 h and 8 h with different CNP concentrations. A total of 47–48 oocytes were stained per condition (4 replicates). (B) GV rate of oocytes cultured for 6 h and 8 h with different CNP concentrations plus estradiol. A total of 46–50 oocytes were assessed per condition (4 replicates). Each bar represents mean + SEM. Different superscript letters (a-c) in each column indicate statistically significant differences (P<0.05). (C) Relative gene expression of natriuretic peptide receptor (NPR2) in COCs after 6 h of pre-IVM with CNP and E2. Five samples were tested per group (10 COCs per sample). Each bar represents relative quantification (RQ), and error bars show RQ max and RQ min. Superscript symbols indicate statistical differences relative to control 0 h: (*) P<0.001; (**) P<0.0001.

**Fig 2:**
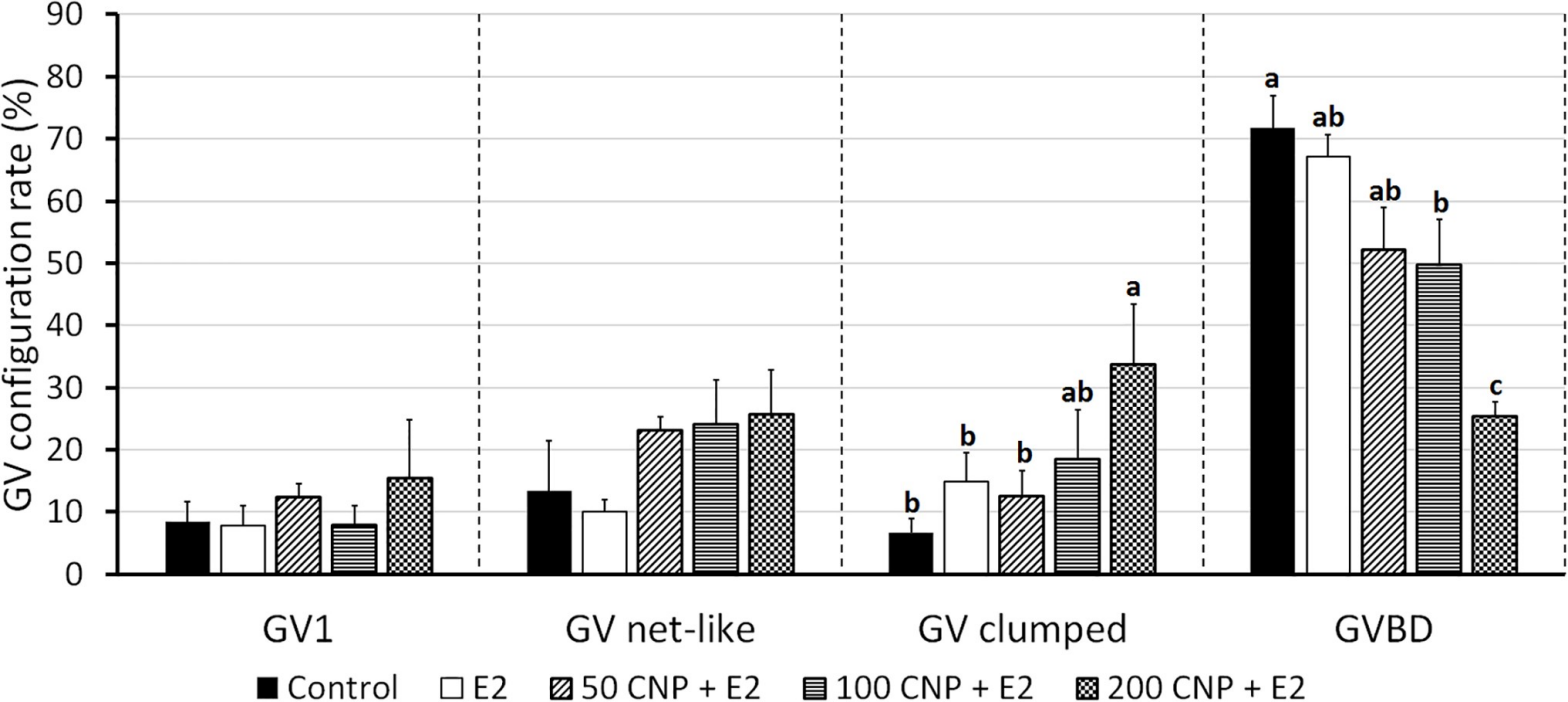
Effect of pre-IVM with CNP and estradiol for 6 h on germinal vesicle chromatin configuration of juvenile-goat oocytes. Germinal vesicles (GV) are classified as GV1: diffuse filamentous chromatin; GV net-like: condensed net-like chromatin; GV clumped: condensed clumped chromatin; GVBD: broken nuclear membrane. A total of 46–50 oocytes per treatment were assessed (4 replicates). Each bar represents mean + SEM. Different superscript letters (a-c) in each column indicate statistically significant differences (P<0.05).

In experiment 1 (Fig 1A), the effect of various CNP concentrations (0, 50, 100 and 200 nM) on oocyte meiotic arrest was evaluated. No differences were found among treatments. In experiment 2 (Fig 1B), the same CNP concentrations were tested combined with 10 nM E2. Pre-IVM with 200 nM CNP + E2 maintained oocytes at GV stage at a higher rate than control group for 6 h (75% vs 28%; P<0.05). After 8 h, there were no differences amongst treatments and the rate of oocytes at GV in the 200 nM + E2 group decreased (P<0.05). In experiment 3 (Fig 1C), *NPR2* expression was quantified after pre-IVM with or without E2, compared to uncultured COCs (control 0 h). There was a decreased expression after 6 h of pre-IVM with CNP (P<0.0001) and CNP + E2 (P<0.001). There was a trend to an increased expression in CNP + E2 group compared to CNP (P = 0.113). In experiment 2, chromatin configuration was assessed after 6 h of pre-IVM (Fig 2). There was a higher rate of oocytes at GV stage with condensed clumped chromatin in the 200 nM CNP + E2 group compared to control group (P<0.05).

### Pre-IVM with C-type natriuretic peptide and estradiol maintains cumulus-oocyte communication (experiment 4)

**Fig 3:**
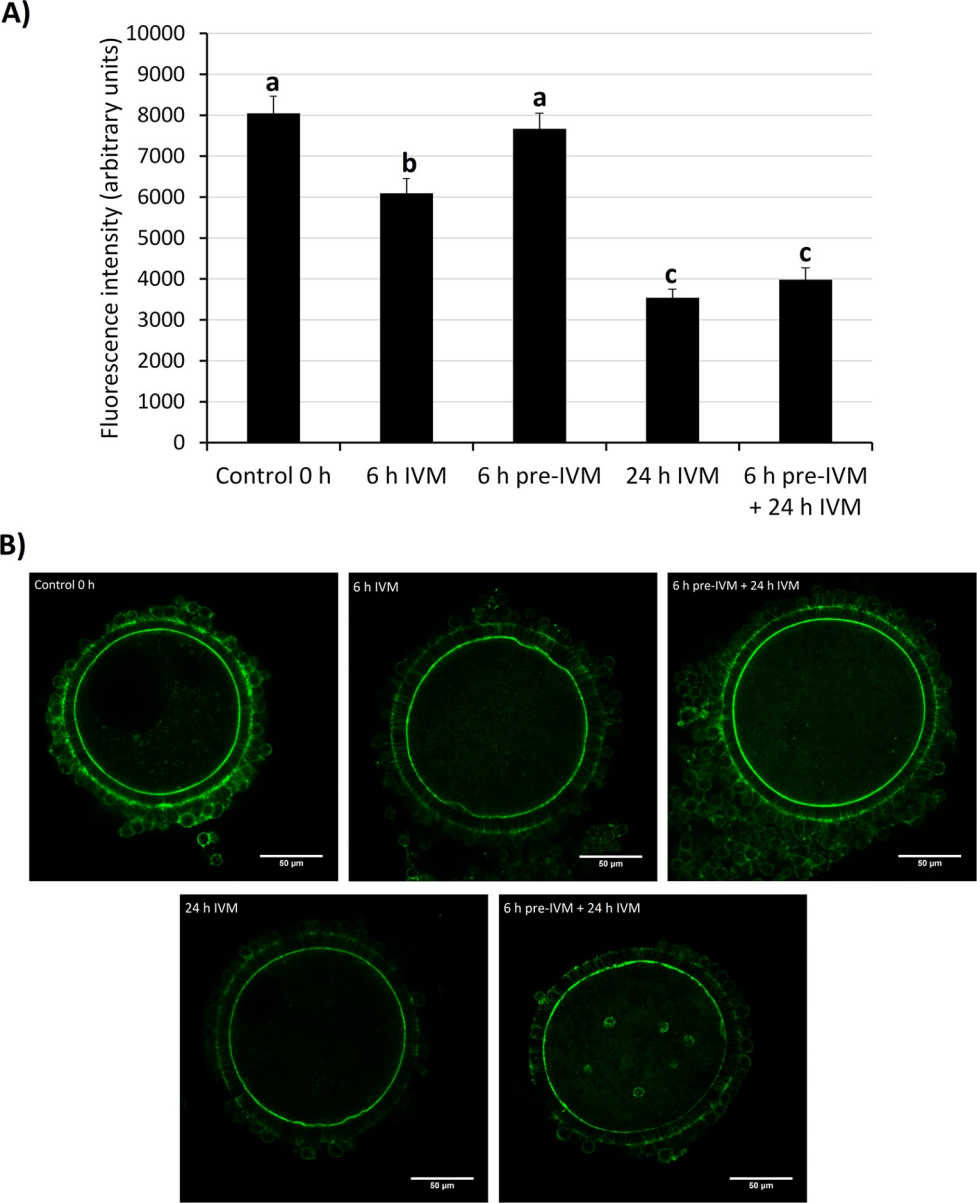
Effect of pre-IVM with CNP and estradiol on the transzonal projections (TZPs) density of juvenile-goat COCs. (A) Phalloidin-FITC Average fluorescence intensity in the zona area of COCs after recovery (control 0 h), 6 h of IVM, 6 h of pre-IVM with CNP and E2, 24 h of IVM, and 6 h of pre-IVM followed by 24 h IVM. At least 32 COCs were assessed per group (4 replicates). Each bar represents mean + SEM. Different superscript letters (a-c) in each column indicate statistically significant differences (P<0.05). (B) Representative confocal images. Positive actin filaments are observed as continuous filaments going from the cumulus cells to the oocyte through the zona region.

In experiment 4 (Fig 3), the effect of pre-IVM with CNP and E2 on CC-oocyte communication was evaluated, compared to conventional IVM. After pre-IVM, COCs presented the same density of TZPs than uncultured COCs (control 0 h), whereas, after 6 h of IVM, the density decreased (P<0.05). At the end of IVM there were greatly fewer TZPs relative to control 0 h, 6 h of pre-IVM and 6 h of IVM (P<0.001).

### Biphasic IVM enhances the oocyte antioxidant defenses and up-regulates the expression of maturation-related genes, but has no effect on nuclear maturation and mitochondria DNA copy number (experiment 5)

**Fig 4:**
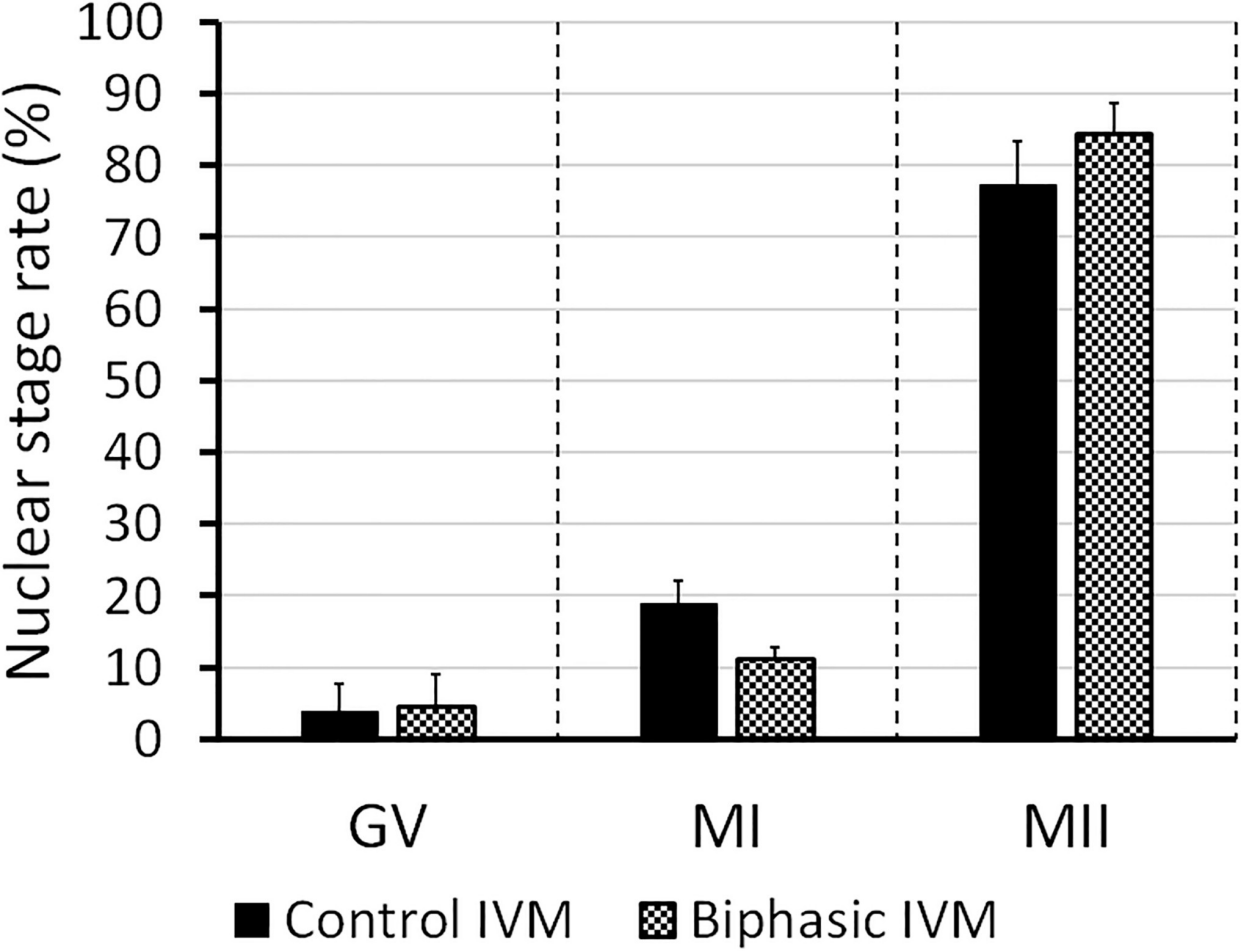
Nuclear maturation rate of juvenile-goat oocytes after biphasic IVM (6 h pre-IVM with CNP and estradiol, followed by 24 h IVM) and control IVM (24 h). Nuclear stage was classified as GV: germinal vesicle; MI: metaphase I; MII: metaphase II. A total of 40 oocytes were assessed per group (4 replicates). Each bar represents mean + SEM.

**Fig 5:**
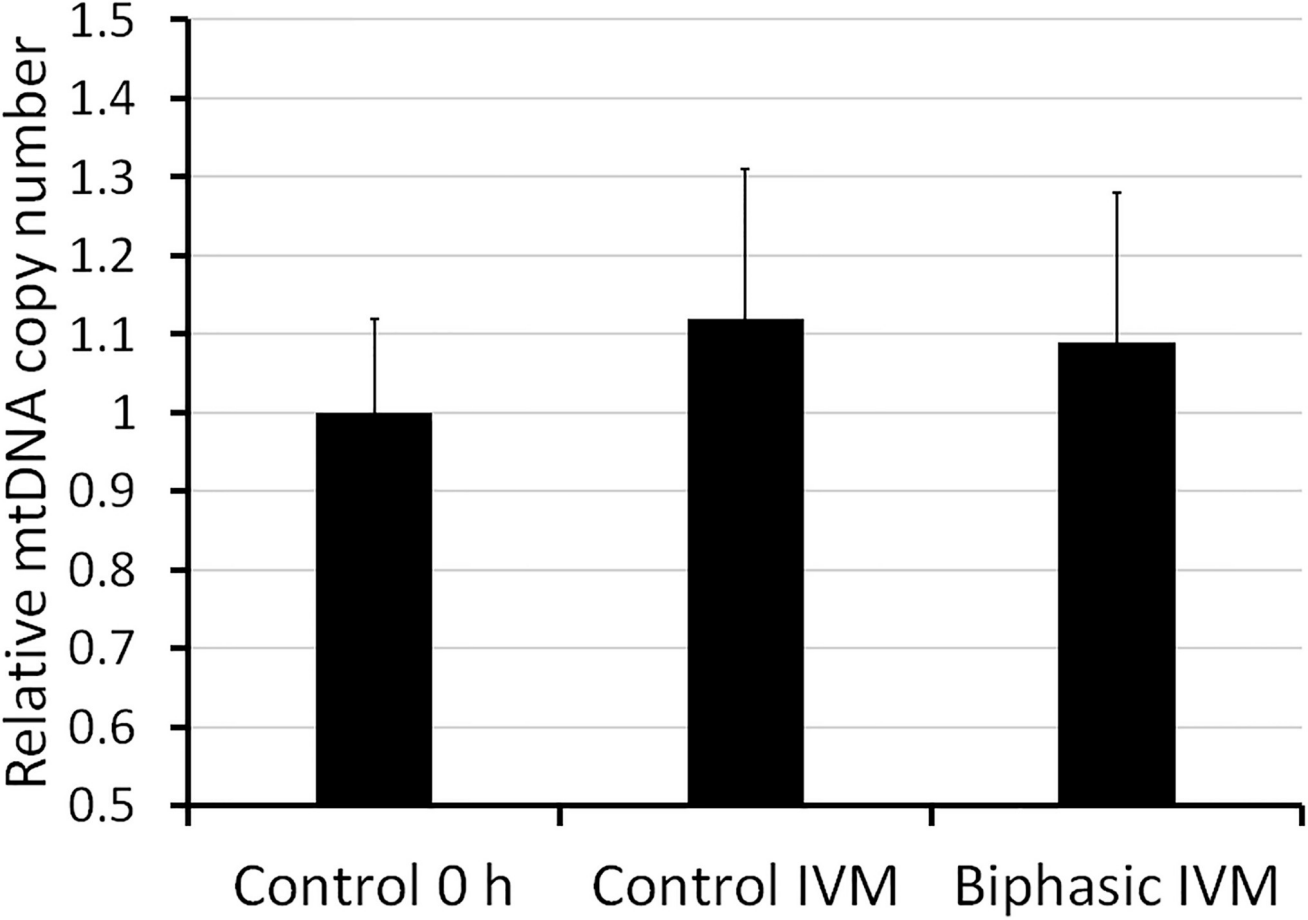
Mitochondrial DNA copy number in juvenile-goat oocytes after recovery from the follicle (control 0 h), biphasic IVM (6 h pre-IVM with CNP plus estradiol, followed by 24 h IVM) and control IVM (24 h). A total of 30 oocytes per group were assessed (3 replicates). Each bar represents mean + SEM.

**Fig 6:**
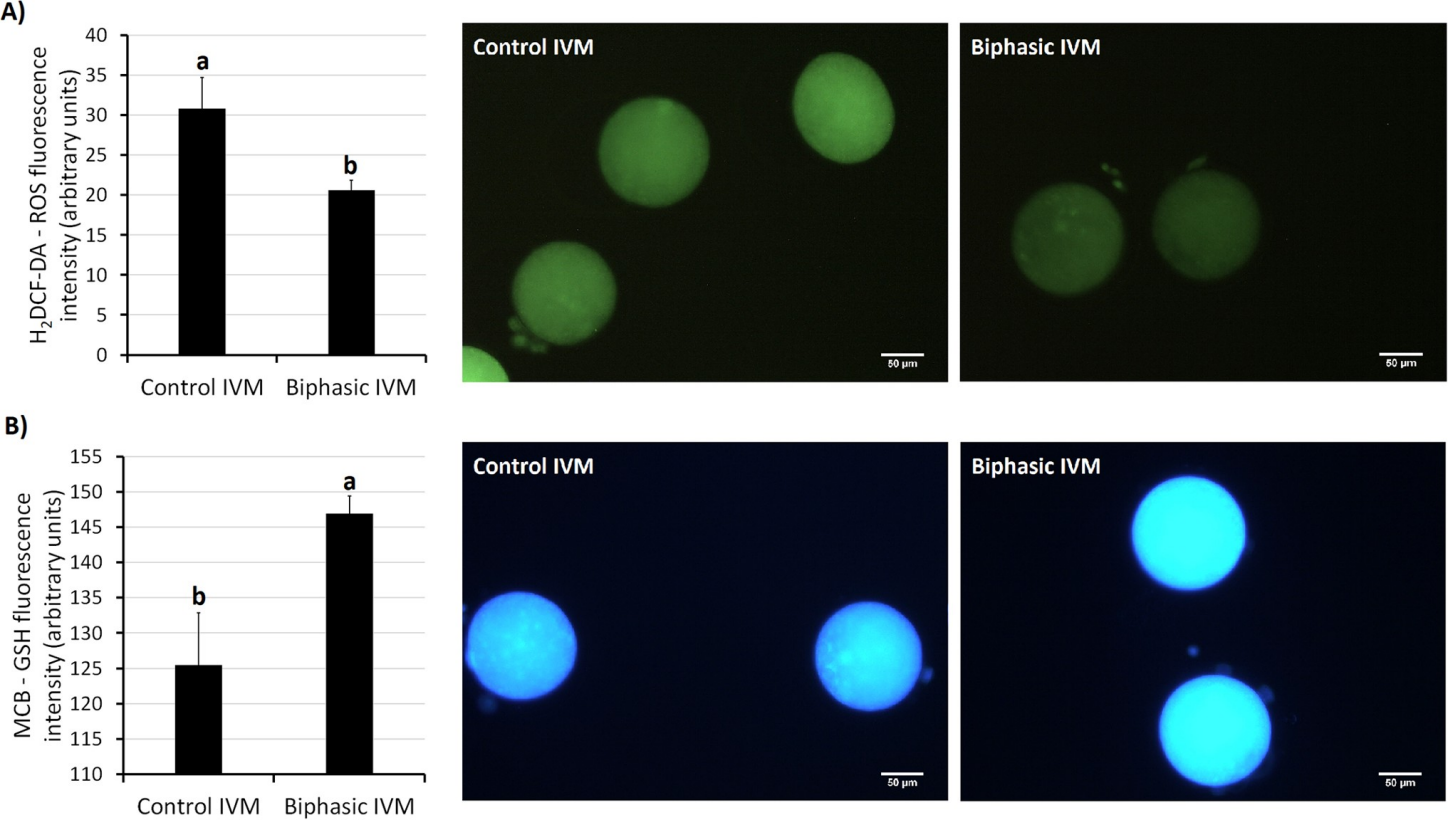
Effect of biphasic IVM on GSH and ROS levels of juvenile-goat oocytes. (A) H_2_DCF-DA-ROS average fluorescence intensity per oocyte after biphasic IVM (6 h pre-IVM with CNP and E2, followed by 24 h IVM) and control IVM (24 h) and representative images. (B) MCB-GSH average fluorescence intensity per oocyte following biphasic and control IVM and representative images. A total of 30 oocytes were assessed per group (3 replicates). Each bar represents mean + SEM. Different superscript letters (a, b) in each column indicate statistically significant differences (P<0.01).

**Fig 7:**
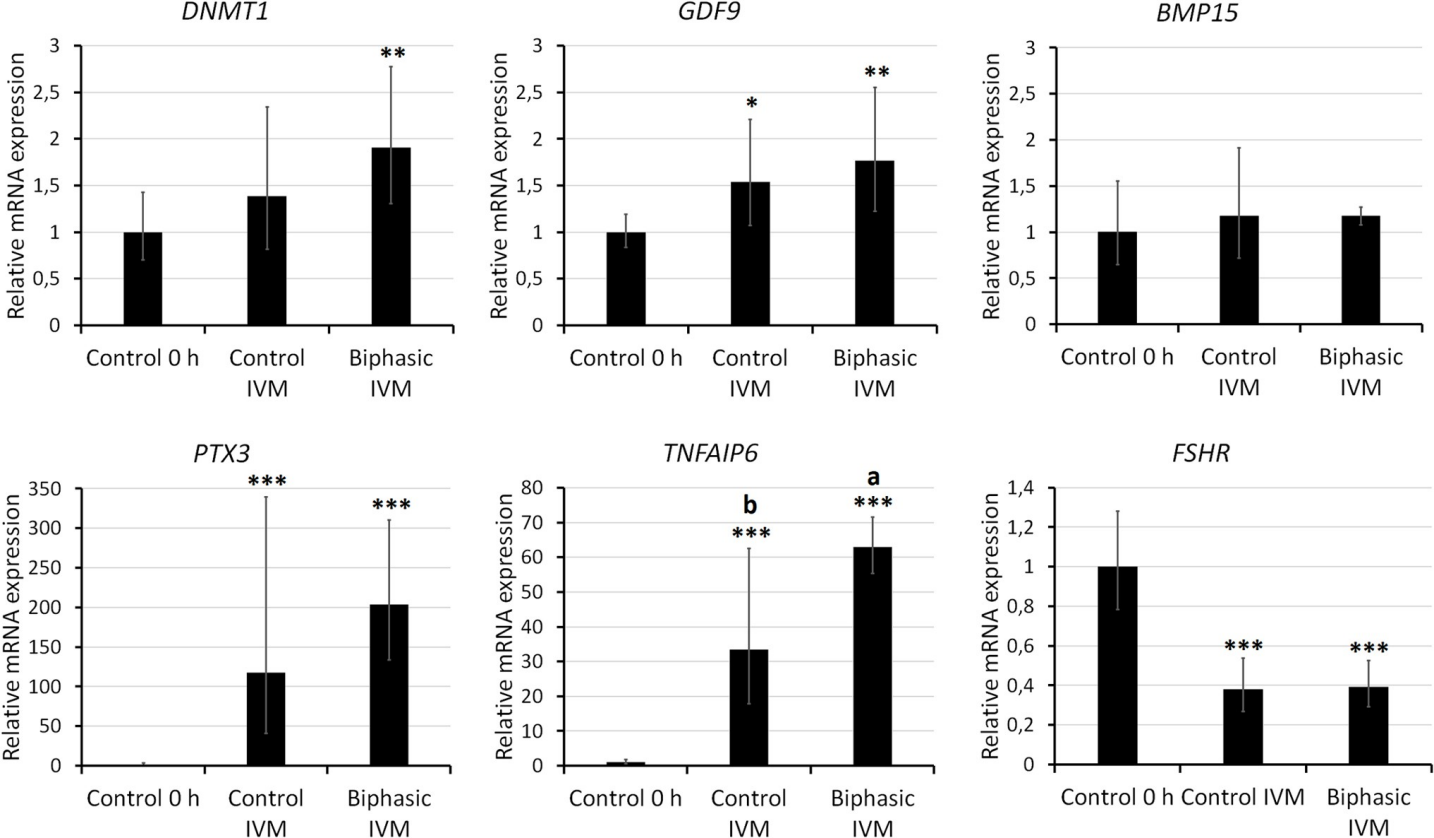
Relative gene expression of *BMP15*, *DNMT1*, *GDF9*, *FSHR*, *PTX3* and *TNFAIP6* in juvenile-goat COCs after biphasic IVM (6 h pre-IVM with CNP and E2, followed by 24 h IVM), control IVM (24 h), and oocyte recovery (control 0 h). Five samples were tested per group (10 COCs per sample). Each bar represents relative quantification (RQ), and error bars show RQ max and RQ min. Superscript symbols indicate statistical differences relative to control 0 h: (*) P<0.05; (**) P<0.001; (***) P<0.0001. Different superscript letters (a—b) indicate statistical differences between treatment groups (P<0.05).

In experiment 5, we assessed various parameters at the end of biphasic IVM and control IVM. Assessment of nuclear stage (Fig 4) showed that the rate of oocytes at MII stage was approximately 80% for both treatments. Neither biphasic IVM nor control IVM modified the mtDNA copy number (Fig 5) after oocyte recovery (control 0 h). As shown in Fig 6, biphasic IVM enhanced intra-oocyte GSH levels compared to control IVM (P<0.001) and decreased intra-oocyte ROS levels (P<0.001). Lastly, COC expression of target genes was evaluated compared to uncultured COCs (control 0 h; Fig 7). Both biphasic and control IVM up-regulated *GDF9* (*P* < 0.05), but only biphasic IVM up-regulated *DNMT1* (P<0.01). Both *PTX3* and *TNFAIP6* were up-regulated after biphasic IVM and control IVM (P<0.0001). But, *TNFAIP6* was more enhanced in the biphasic IVM group than the control IVM group (P<0.05). *FSHR* was down-regulated in both control and biphasic IVM (P<0.0001).

### Biphasic IVM enhances embryo development (experiment 6)

**Fig 8:**
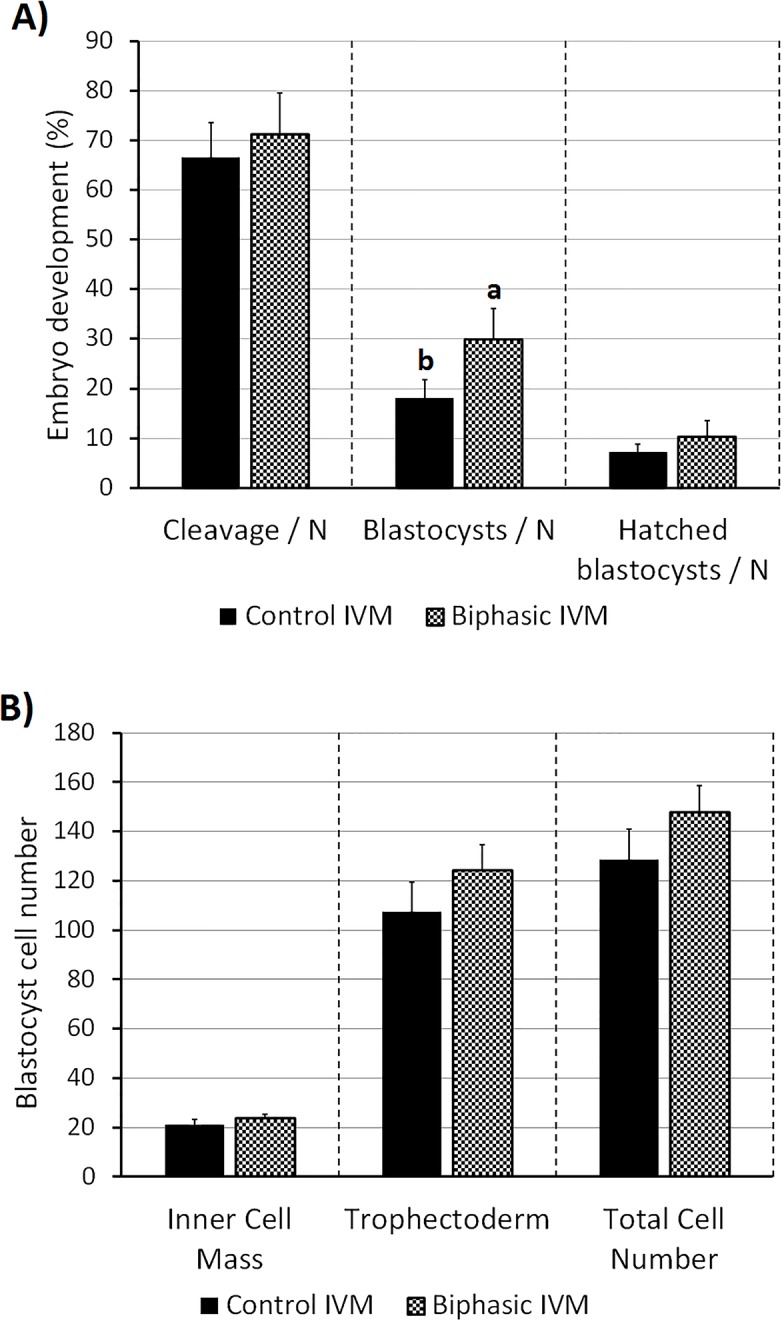
Blastocyst development and quality of juvenile-goat oocytes after biphasic IVM (6 h pre-IVM with CNP plus E2, followed by 24 h IVM) and control IVM (24 h). (A) Cleavage rate (48 hpf) and blastocyst rate (8 dpf) per number of oocytes (N). A total of 196–198 oocytes were cultured per group (5 replicates). (B) Cell count of the inner cell mass and the trophectoderm of expanded and hatched blastocysts. A total of 22–33 blastocysts were analyzed per group (4 replicates). Each bar represents mean + SEM. Different superscript letters (a-b) in each column indicate statistically significant differences (P<0.01).

In experiment 6 (Fig 8), oocytes were in vitro fertilized and embryo cultured for 8 days after biphasic IVM and control IVM. Oocytes cultured in the biphasic IVM system yielded more blastocysts when compared to control IVM (29.9% vs. 18.1%; P<0.01). No differences were observed in the blastocyst cell number or cell allocation.

## Discussion

In the present study we aimed to improve IVEP in juvenile goats by implementing a biphasic IVM system that consists of a pre-IVM phase with CNP followed by standard IVM. First, we developed a pre-IVM phase with CNP and estradiol that sustained meiotic arrest and cumulus-oocyte communication for 6 h. Second, we evaluated the effect of biphasic IVM on oocyte embryo developmental competence.

Pre-IVM with 200 nM CNP delayed GVBD when combined with estradiol. Previous studies have already shown that CNP sustains meiotic arrest for 6 h in cattle [10,13,15,32], sheep [16] and adult goat [17], and for at least 24 h in mouse [18] and human [19]. In our study estradiol was essential for enabling CNP to avoid GVBD. Similarly estradiol prolonged CNP meiotic arrest from 4 to 6 h in adult goats [17] and from 24 to 48 h in juvenile mice [18]. This is related to estradiol promoting CNP receptor (NPR2) in COCs, which was first shown in mouse [42], and also observed in bovine cumulus cells and oocytes [10]. In our study, *NPR2* considerably decreased after 6 h of pre-IVM, despite the presence of estradiol, although there was a trend to a lower decline compared with pre-IVM without estradiol. This lower *NPR2* expression could explain the transient effect of CNP on meiotic arrest, which is not maintained for 8 h. According to previous studies, *NPR2* expression decreases in a time-dependent manner after the COC is retrieved from the follicle and is related to the GVBD [43]. Oocyte secreted factors (OSFs), such as GDF9 and BMP15, can also up-regulate *NPR2* [10], hence could further prolong meiotic arrest during pre-IVM.

Pre-IVM with CNP and estradiol increased the proportion of GVs with condensed clumped chromatin, which suggests an improved oocyte competence. During follicular development, chromatin changes progressively from disperse to condense configurations related to acquisition of meiotic and developmental competence [44,45]. Pre-IVM with CNP also induced condensed chromatin configurations in mice (surrounded nucleolus) [18] and cattle (GV2) [13].

For determining if the pre-IVM period could also sustain cumulus-oocyte communication, TZP density was assessed. TZPs are actin filaments arising from cumulus cells that traverse the zona pellucida to the oocyte [46], which form GJs with the oolemma of the oocyte (reviewed by Russell et al. [47]). In our study, there was a progressive decrease in TZP density during IVM. But pre-IVM maintained TZP density for 6 h after follicular recovery, in accordance to previous results in mice [18] and humans [48]. Pre-IVM with IBMX plus forskolin also prevents the loss of GJC [21,22]. This enhanced cumulus-oocyte communication at 6 h could have a positive impact on the acquisition of oocyte competence. It is reported that TZPs enable the exchange of cell signaling molecules during follicular development (reviewed by Albertini et al. [49]), including the transfer of mRNA and metabolites [46,50].

Based on these results, for our further experimentation we compared two IVM systems: biphasic IVM (6 h pre-IVM with CNP plus estradiol, followed by 24 h IVM) and control IVM (24 h). We evaluated different parameters that indicate oocyte competence: nuclear maturation, mtDNA copy number, GSH and ROS levels, and COC gene expression. The assessment of nuclear maturation showed that around 80% of oocytes reached MII at the end of biphasic and control IVM systems. However, when applied to adult goat COCs, biphasic IVM with CNP increased the rate of oocytes reaching MII over controls [17]. In our study, we have not observed this effect possibly due to the already high proportion of MII-oocytes in the control group.

The oocyte mtDNA copy number is correlated to the number of mitochondria and is a marker of oocyte competence (reviewed by Fragouli & Wells [51]). The mitochondria number increases during folliculogenesis [52], but is stable during oocyte maturation and early embryo development [53]. Hence, we hypothesized that a pre-IVM, which simulates the latest phase of follicular development, will increase the mtDNA copy number, as previously reported in cattle [10,15]. However, we did not observed this effect in our study, which could be related to a different response of goat oocytes to CNP or to different specific pre-IVM culture conditions between studies. Other mitochondria analysis could show different effects of biphasic IVM in goat oocytes, such an increase of mitochondrial activity, as observed in sheep [16].

Biphasic IVM increased intra-oocyte GSH levels and decreased ROS compared to control IVM. ROS-induced oxidative stress impairs maturation and embryo development (reviewed by Tamura et al. [54]), whereas GSH is a significant non-enzymatic antioxidant for the oocyte (reviewed by Guérin et al. [55]) and promotes male pronucleus formation after IVF [56]. In mouse and cow oocytes, the increase in GSH levels after pre-IVM with IBMX plus forskolin was associated with the maintenance of GJC that enables GSH transfer from CC [21,22]. Our results suggest a similar mechanism in juvenile-goat oocytes which is promising for improving JIVET. The low oocyte competence in juvenile females is related to a higher exposure to ROS due to impaired GSH synthesis [25]. In our laboratory, we observed that juvenile-goat oocytes have lower GSH concentration than adults, and increasing GSH by supplementing IVM medium with cysteamine leads to higher blastocyst yield [24].

Lastly, biphasic IVM up-regulated *DNMT1* in juvenile-goat COCs. DNMT1 is the major methyltransferase in bovine oocytes [57]. As reviewed by Uysal & Ozturk [38], DNA methylation by DNMTs increases during follicular development and oocyte maturation and is essential for early embryo development. An up-regulation of extracellular matrix-related genes (*TNFAIP6* and *PTX3*) after IVM was also observed, which was further enhanced after biphasic IVM compared to control IVM. These proteins are usually down-regulated in vitro, consequently impairing oocyte competence (reviewed by Brown et al. [41]). Sugimura et al. [58] reported that pre-IVM with IBMX and dbcAMP enhances the expression of *TNFAIP6* and other extracellular matrix genes in bovine CC. Similarly to the Sugimura et al. study, there was a drastic decrease in *FSHR* expression after IVM regardless of the culture system. FSH is essential for acquiring oocyte competence during follicular development prior to maturation [59] and the expression of its receptor is a predictor of oocyte quality (reviewed by Ferreira et al. [40].

Overall, our results suggest that biphasic IVM improves juvenile-goat oocyte competence. This was further confirmed by assessing embryo development: oocytes cultured with biphasic IVM produced more blastocysts than control IVM. An improvement in blastocyst development has been previously reported in cattle [10,13,15], sheep [16], pig [60] and mouse [18] after IVF, and in adult goat after parthenogenetic activation [17]. In cattle, an extended IVM phase (26–28 h) is needed to improve blastocyst rate after pre-IVM with CNP [10], whereas a 24-h IVM only induces an effect on blastocyst quality [13]. However, in Zhang et al.[16], a sheep COC IVM phase of 24 h enhanced the blastocyst rate, while 28 h had a detrimental effect. This study, and our results, suggest that the biphasic IVM system with a conventional IVM period is efficient in small ruminants.

Despite this, the blastocyst production is only slightly improved in ruminants, whereas juvenile-mice oocytes developed to similar blastocyst rate compared to IVEP of ovulated oocytes [18]. The shorter meiotic arrest in ruminants, compared to 24–48 h in mice, possibly accounts for this difference. Sasseville et al. [61] reported that bovine cumulus cells have a predominant PDE8 activity, which suggests that other PDE inhibitors may be needed to efficiently arrest oocyte meiosis in ruminants. For instance, Santiquet et al. [62] were able to sustain meiotic arrest in bovine oocytes for 21 h with a combination of CNP, estradiol, cilostamide (a PDE3 inhibitor) and sildenafil (a PDE5 inhibitor). Furthermore, other medium components that can improve oocyte quality during pre-IVM should be considered. In mice the significant improvement in blastocyst development is only achieved when FSH and GDF9 are included in the pre-IVM with CNP [18].

## Conclusions

In conclusion, a pre-IVM that incorporates CNP and estradiol inhibits meiotic resumption for 6 h in juvenile-goat oocytes while maintaining cumulus-oocyte communication. This pre-IVM followed by standard IVM in a biphasic IVM system improves the oocyte protection against oxidative stress, up-regulates genes related to DNA methylation and extracellular matrix formation and enhances blastocyst development. This study shows that biphasic IVM provides additional developmental competence to juvenile-goat oocytes and is a promising procedure for improving IVEP with low-quality oocytes. Future experiments should focus in prolonging the temporary meiotic arrest and refining the pre-IVM medium with FSH and OSFs, which could further enhance oocyte competence.

## Supporting information

S1 Dataset Data from experiment 1. Frequency table of germinal vesicle.
(XLSX)

S2 Dataset Data from experiment 2. Frequency table of germinal vesicle.
(XLSX)

S3 Dataset Data from experiment 3. Results of RT-qPCR: (1) amplification, and (2) relative quantification.
(XLSX)

S4 Dataset Data from experiment 4. Results of mean fluorescence intensity of phalloidin-FITC from transzonal projections.
(XLSX)

S5 Dataset Data from experiment 5. (1) Frequency table of nuclear stage; results of mean fluorescence intensity of (2) H_2_DCF-DA (ROS) and (3) MCB (GSH); (4) results of mtDNA copy number; and results of RT-qPCR: (5) amplification and (6) relative quantification.
(XLSX)

S6 Dataset Data from experiment 6. (1) Frequency table of embryo development and (2) results of blastocyst cell number.
(XLSX)
